# Safe definitive surgery in unstable pelvic ring injuries – a retrospective cohort study

**DOI:** 10.1007/s00590-026-04972-y

**Published:** 2026-09-23

**Authors:** Carl Vincent Bästlein, Felix Karl-Ludwig Klingebiel, Christian Thomas Hübner, Yannik Kalbas, Olivia Klee, Hans-Christoph Pape, Roman Pfeifer

**Affiliations:** University Hospital of Zurich, Zurich, Switzerland

**Keywords:** Unstable pelvis, percutaneous sacroiliac screw, safe definitive surgery, pelvic ring stabilization

## Abstract

**Introduction:**

Unstable pelvic ring injuries in polytrauma patients remain among the most lethal injury patterns, largely due to hemorrhage and the physiological burden of staged surgical management. While temporary external fixation is widely used as part of damage-control strategies, early definitive posterior stabilization using percutaneous sacroiliac “rescue screws” may provide sufficient mechanical stability while reducing cumulative operative stress. However, comparative clinical data supporting this approach remain scarce. This study investigates operative strategies, timing, and early outcomes in a consecutive cohort of severely injured patients with unstable pelvic ring fractures treated at a Level-1 trauma center.

**Methods:**

We performed a retrospective cohort study including patients aged ≥ 16 years with high-energy unstable pelvic ring injuries (Young and Burgess APC II–III, LC III, combined mechanism, and vertical shear) and an Injury Severity Score (ISS) > 16. Demographic data, injury severity, hemodynamic status on admission, operative strategies, number of surgical procedures, and length of hospital stay were analyzed. Outcomes were compared between early definitive posterior fixation and temporary external fixation. Statistical significance was defined as *p* < 0.05.

**Results:**

Seventy-two patients were included (mean age 43.6 ± 17.3 years; 72.2% male), reflecting a severely injured cohort with a mean ISS of 30.4 ± 10.6; 30.6% presented with hemodynamic shock on admission. Early definitive posterior stabilization without prior temporary external fixation was achieved in *n* = 63 patients (87.5%). Temporary external fixation was required in *n* = 9 patients (12.5%), who demonstrated significantly greater injury severity, including higher ISS (41.0 ± 11.3 vs. 28.9 ± 9.8), higher NISS (45.7 ± 12.5 vs. 30.8 ± 10.4), and a higher incidence of shock on admission (44.4 versus. 28.6%). Compared with early definitive fixation, external fixation was associated with a substantially higher operative burden (2.31 ± 0.85 vs. 1.41 ± 0.67 procedures) and prolonged hospitalization (31.9 ± 30.2 vs. 23.4 ± 25.3 days). Radiographic reduction quality did not differ between the groups, with a mean residual sacroiliac joint diastasis of 4.14 ± 1.21 mm in the SDS group and 4.11 ± 0.71 mm in the ExFix group; according to the Matta and Saucedo criteria, all reductions in both groups were graded as anatomic or nearly anatomic. Importantly, early posterior fixation using percutaneous sacroiliac rescue screws was not associated with an increase in early fixation-related complications, even in initially unstable patients.

**Conclusion:**

In a Level-1 trauma setting, early definitive posterior stabilization using percutaneous sacroiliac rescue screws is feasible and safe in most patients with unstable pelvic ring injuries. Compared with traditional staged management using temporary external fixation, this strategy was associated with a reduced cumulative operative burden and a shorter hospital length of stay without compromising early safety. Given the small number of patients managed with external fixation, these comparative findings should be interpreted with caution. External fixation should be reserved for select patients with extreme injury severity or specific limiting factors for safe definitive surgery. These findings question the routine use of staged damage-control strategies and support early posterior definitive fixation as a component of modern pelvic trauma care.

## Introduction

 High-energy pelvic trauma is a critical condition that frequently involves multi-system injuries, necessitating a multidisciplinary medical approach [1]. These injuries are among the most severe cases in trauma surgery, carrying a high risk of mortality and life-threatening hemorrhage and instability [2]. Especially in polytraumatized patients, the presence of a severe or unstable pelvic ring injury is associated with high Injury-Severity Score (ISS) values and consequently high mortality rates [2, 3]. Unstable pelvic ring fractures are among the most severe trauma cases because they pose a significant risk of exsanguination due to massive bleeding [1, 2]. While hemorrhage is the immediate threat, sepsis, multiple organ failure and local infections, especially in open pelvic fractures, are significant causes of later mortality [4]. Data from long-term trauma databases show that associated conditions like pneumonia and septic shock contribute to the overall mortality rate of polytraumatized patients with pelvic injuries. External fixation (ExFix) plays a central and historically significant role in the initial management of hemodynamically unstable pelvic ring fractures. It remains the most frequently documented and utilized technique for temporary pelvic stabilization globally [2, 5]. The primary role of external fixation is to provide rapid mechanical stabilization of the pelvic ring to reduce intrapelvic cavity volume. This reduction facilitates intrapelvic clot formation and tamponade of venous bleeding, which is critical for hemorrhage control in the acute phase [5]. However, external fixation is often insufficient in vertical shear fractures, as it primarily controls the anterior ring and may not provide enough stability to the posterior pelvic ring. Common risks include pin tract infections (reported in up to 19% of cases), loosening of the pins, and limited feasibility in obese patients. It can be cumbersome for nursing care and may interfere with certain surgical approaches. Similarly, the C-clamp was introduced to provide emergency stabilization of the posterior pelvic ring; however, its use has seen a notable decline over recent decades due to a high rate of serious complications and technical difficulties [3, 5]. Both external fixation and C-Clamp are generally a temporary measure; survivors usually require a second “conversion surgery” to replace the fixator with definitive internal hardware.

The literature provides numerous percutaneous fixation techniques for definitive care [3, 5, 6].

These percutaneous techniques enable early definitive stabilization of the anterior and posterior pelvic ring while minimizing cumulative surgical burden, thereby supporting faster recovery in severely injured patients by reducing the physiological load associated with multiple extensive procedures.

This retrospective cohort study aims to present our management strategy for severely injured patients with unstable pelvic ring injuries at a Level-1 trauma center. In particular, this study demonstrates that the use of percutaneous screws, even in complex fracture patterns and hemodynamically unstable patients, enables early definitive stabilization without the need for an external fixator.

## Materials and methods

A retrospective cohort study of trauma patients with unstable (Young & Burgess APC II–III, LC III, CM, VS) pelvic ring injuries at a level one trauma center was performed. Patients > 16 years, with an ISS > 16 and injured by a high energy mechanism were included. Exclusion criteria were pathologic fracture, low energy trauma and missing consent. Collected variables included demographics, ISS, shock on admission, LOS (length of stay), number of operations, revisions and operative strategy (ExFix vs. Non-ExFix). Comparative analyses were performed between patients managed with external fixation versus definitive internal fixation. Statistical significance was set at *p* < 0.05. 

### Setting

The study was conducted in accordance with the Declaration of Helsinki and approved by the Swiss Cantonal Ethics Committee (*Approval number removed for blinding*). The study was performed in a Swiss level 1 trauma center. All consecutive patients receiving SI screw fixation from November 2014 to December 2021 were screened for eligibility.

All participants gave written consent for the use of retrospective data for scientific research. 

### Participants

Each patient who underwent primary percutaneous screw fixation during the recruitment period was screened for eligibility. Patient identification was performed via manual review of surgical records by two authors and an additional automated search of the clinical information system.

### Study groups and definitions

Patients were stratified according to the initial operative strategy for pelvic ring stabilization. The primary comparison was performed between patients treated with early definitive fixation using percutaneous screws (Safe Definitive Surgery group, SDS) and those managed with temporary external fixation (ExFix group) as part of a staged damage-control approach.

The SDS group was defined as patients who underwent early definitive stabilization of the pelvic ring using percutaneous SI screws without prior temporary external fixation. This approach reflects the concept of safe definitive surgery.

The ExFix group included patients who received temporary external fixation as the initial stabilization strategy. This group was further subdivided into patients treated with external fixation alone, and patients undergoing combined external fixation and early percutaneous screw fixation during the same initial operative procedure.

Unstable pelvic ring injuries were defined based on fracture patterns associated with posterior instability and significant ligamentous disruption. According to the Young and Burgess classification, these included anterior–posterior compression type II–III (APC), lateral compression type III (LC), vertical shear (VS) and combined mechanism injuries (CM).

High-energy trauma was defined as injury mechanisms such as motor vehicle collisions, falls from height (> 3 m), or high-impact trauma. Low-energy trauma (e.g., fall from standing height) was excluded.

Hemodynamic shock on admission was defined as a systolic blood pressure < 90 mmHg and/or the requirement for vasopressor support.

External fixation as a damage-control strategy was defined as a temporary stabilization procedure performed in physiologically unstable patients or when definitive posterior fixation was not immediately feasible due to clinical or logistical constraints.

### Outcomes variables

Radiographic reduction quality of the posterior pelvic ring was assessed by measuring the residual diastasis of the sacroiliac joint on postoperative imaging. Reduction was graded according to the criteria described by Matta and Saucedo, with a residual displacement of less than 4 mm classified as anatomic, 4–10 mm as nearly anatomic, 11–20 mm as moderate, and more than 20 mm as poor [7]. Residual sacroiliac joint diastasis was compared between the SDS group and the ExFix only group.

The total number of surgical procedures (OP count) was defined as all surgical interventions performed during the index hospitalization, irrespective of anatomical region or indication, including staged anterior ring fixation procedures. Revision was defined as any unplanned return to the operating room directly attributable to the posterior pelvic fixation, including hardware-related complications and inadequate reduction. Planned hardware removal at the time of fracture consolidation and staged anterior ring procedures were not counted as revisions.

### Data extraction

The data were organized and stored using Microsoft Excel on password-protected in-house computers. The missing data rate was extremely low, as parameters of interest were defined a priori with special focus on the ones that were routinely inserted in our clinical system. In case of missing data, the retrospective parameter for this patient was marked as N/A (not available) and excluded from analysis.

### Data analysis

Continuous data are presented with mean and standard deviation, categorical variables with numbers and percentages. Statistical analysis was performed in R using the “Stat” and “Tableone” packages. Figures were computed using the “ggplot2” package. MS-Excel was used for data visualization. Data were visually tested for normality using histograms. Binary data were assessed using a two-sided Fisher’s exact test, non-binary categorical data using the chi-squared test with Yates’ correction for continuity, and continuous parameters with the t-test or Mann–Whitney U test. Significance levels were set at 0.05.

## Results

A total of 72 patients identified between 11/2014 and 12/2021 with unstable pelvic ring injuries met the inclusion criteria. The mean age of the cohort was 43.6 ± 17.3 years, and 52 patients (72.2%) were male. Overall injury severity was high, with a mean ISS of 30.4 ± 10.6 and a mean NISS of 32.7 ± 11.8. Hemodynamic shock on admission was present in 22 patients (30.6%). (Table 1)

**Table 1:** Overview

-----	Overall	APC	CM	LC	VS
N	72	17	13	15	27
Age (mean (SD))	43.57 (17.33)	54.41 (15.10)	35.00 (12.48)	44.07 (19.26)	40.59 (16.90)
Sex (= M(%))	52 (72.2)	17 (100.0)	10 (76.9)	8 (53.5)	17 (63.0)
ISS (mean (SD))	30.44 (10.61)	27.47 (11.68)	32.15 (10.47)	28.60 (9.53)	28.60 (9.53)
NISS (mean (SD))	32.67 (11.48)	28.41 (11.88)	35.00 (10.50)	30.33 (9.19)	35.52 (12.28)
Shock (= yes (%))	22 (30.6)	3 (17.6)	3 (23.1)	6 (40.0)	10 (37.0)

The cohort was stratified into three treatment groups based on the initial pelvic stabilization strategy: (1) Safe Definitive Surgery (SDS) - primary percutaneous posterior fixation without temporary external fixation; (2) ExFix + Dorsal Stabilization - initial external fixation combined with simultaneous definitive posterior fixation; and (3) ExFix only - temporary external fixation as the sole initial stabilization method.

Primary definitive percutaneous stabilization without temporary external fixation was performed in 63 patients (87.5%) (SDS group). Temporary external fixation as part of an initial damage-control strategy was required in 9 patients (12.5%), of whom 4 patients (5.6%) underwent initial combined stabilization with external fixation and definitive posterior fixation using percutaneous sacroiliac screws in the first surgical procedure (ExFix + Dorsal Stabilization group), and 5 patients (6.9%) were treated with external fixation alone as the initial pelvic stabilization method (ExFix only group). (Table 2)

**Table 2:** Non-ExFix vs. ExFix

	Non-ExFix	Ex-Fix
OP Count (n)	1.41	2.31
LOS (d)	23.39	31.92

Anterior ring fixation was performed simultaneously during the index procedure in *n* = 15 patients (20.8%), as a staged secondary intervention in *n* = 8 patients (11.1%), and managed non-operatively in *n* = 49 patients (68.1%). Rates of simultaneous anterior ring fixation were highest in APC II injuries (70%), consistent with the defining symphyseal disruption in this pattern, and lowest in LC III (6.7%) and vertical shear injuries (14.8%), reflecting mechanically stable or impacted anterior ring components in these fracture patterns.

Patients requiring temporary external fixation showed markedly higher injury severity compared to the SDS group. The ExFix + Dorsal Stabilization group demonstrated a mean ISS of 40.3 ± 12.2 and a mean NISS of 45.0 ± 9.8, with hemodynamic shock on admission present in n 3 patients (75.0%). Combined mechanism injuries according to the Young & Burgess classification were present in 2 patients (50.0%), indicating a substantially more unstable injury pattern. Indications for external fixation in this subgroup were highly case-specific and included an open pelvic ring injury and a combined pelvic ring and acetabular fracture. These individual clinical contexts are summarized descriptively in Table 4. The ExFix only group demonstrated a mean ISS of 41.6 ± 11.1 and a mean NISS of 46.4 ± 14.4, with shock present in 1 patient (20.0%), and combined mechanism injuries in 3 patients (60.0%). In contrast, the SDS group showed a mean ISS of 28.9 ± 9.8 and a mean NISS of 30.8 ± 10.2, with hemodynamic shock on admission in 18 patients (28.6%). (Table 3)

**Table 3:** Comparison SDS vs. ExFix + dorsal stabilization vs. ExFix

	Overall	SDS	ExFix (Damage Control) + dorsal Stabilisation	ExFix	
N	72	63	4	5	
Unstable PI	72	63	4	5	
Age (years)	43.57	45.40	33.75	28.4	
ISS	30.44	28.94	40.25	41.6	
NISS	32.67	30.79	45	46.4	
Shock	22	18	3	1	
CM	13	8	2	3	

Among the 5 patients in the ExFix only group, the reasons for not performing early posterior percutaneous fixation were heterogeneous and predominantly driven by logistical or physiological constraints. In 3 patients (60.0%), stabilization with an external fixator was chosen because no pelvic trauma surgeon was available. One patient (20.0%) arrived in profound hypothermia (31.6 °C), requiring rapid stabilization with minimal operative duration. In the remaining one patient (20.0%), although documentation did not explicitly state the rationale, the extent of polytrauma and multiple concomitant injuries likely favored immediate application of an external fixator. (Table 4 and 5) Overall, these cases illustrate that the use of external fixation was context-specific and driven by situational clinical factors rather than by a deliberate alternative strategy to early safe definitive surgery using percutaneous screws for posterior pelvic ring fixation.

**Table 4:** External Fixation together with definitive dorsal stabilization as first operation

Patient	Pelvic injury	Reason for initial external fixation	Clinical context / notes
1	Open pelvic ring injury	Open pelvic fracture	Morel-Lavallée lesion
2	VS	Damage-Control surgery	Hemodynamic instability
3	VS	Damage-Control Surgery	Severe polytrauma
4	CM	Combined pelvic and acetabular injury	External fixation needs for complex pelvic ring fracture concomitant with an acetabular fracture

The mean residual sacroiliac joint diastasis was 4.14 ± 1.21 mm in the SDS group and 4.11 ± 0.71 mm in the ExFix only group, with no significant difference between the groups. According to the Matta and Saucedo criteria, all reductions in both groups were graded as anatomic or nearly anatomic, with an anatomic result (< 4 mm) achieved in 26 patients of the SDS group and 1 patient of the ExFix only group. No patient in either group demonstrated a moderate or poor reduction (> 10 mm residual displacement).

**Table 5:** First operation with only external stabilization

Patient	Pelvic Injury	Reason for initial external fixation	Clinical context / notes
1	CM	No pelvic trauma surgeon available	Temporary ExFix chosen due to limited specialist availability
2	VS	No pelvic trauma surgeon available	Early learning curve
3	VS	Profound hypothermia on admission (31.6 °C)	Hypothermia contributing to trauma induced coagulopathy
4	LC	Damage Control Surgery	Severe Polytrauma
5	VS	No pelvic trauma surgeon available	Temporary ExFix chosen due to limited specialist availability

Among patients treated with SDS using percutaneous sacroiliac screws secondary surgical intervention related to the posterior fixation was required in seven patients (25.9%). Of these, early secondary surgical intervention within three months occurred in four patients (57.1%), whereas late secondary surgical intervention beyond three months occurred in three patients (42.9%). These revisions comprised: hardware loosening requiring screw exchange (*n* = 2), intraforaminal screw malpositioning without neurological deficit requiring repositioning or removal (*n* = 2), intraforaminal malpositioning with neurological deficit requiring urgent removal (*n* = 1), failed closed reduction requiring conversion to open revision (*n* = 1), and sacroiliac pseudarthrosis requiring conversion to lumbopelvic instrumented fixation (*n* = 1). Overall, 20 patients (74.1%) did not require any revision during the observation period. Among patients treated with early definitive posterior fixation using percutaneous sacroiliac screws, the mean total number of surgical procedures was 1.41 ± 0.67. The mean length of hospital stay in this group was 23.4 ± 25.3 days. No early complications attributable to percutaneous SI screw fixation were observed, including in patients with complex fracture patterns or initial hemodynamic instability.

## Discussion

Unstable pelvic ring injuries carry a high risk of mortality, with rates for patients presenting in hemorrhagic shock historically ranging from 40 to 60% [1]. Patient survival is highly dependent on the timely initiation of hemorrhage control, mechanical stabilization of the unstable pelvic ring and the management of severe concomitant injuries [2].

This retrospective analysis demonstrates that early posterior definitive fixation—frequently performed using percutaneous sacroiliac screws can be safely implemented in a Level-1 trauma setting, even in severely injured or initially unstable patients. Compared with staged management using external fixation, this strategy was associated with a reduced cumulative operative load and may shorten hospitalization without compromising safety. Although the small number of patients treated with external fixation limits direct comparability, these results support the role of early percutaneous posterior stabilization as a viable alternative to traditional damage-control approaches in modern pelvic trauma care.

Early fixation, in this study, was associated with shorter intensive care unit (ICU) and overall hospital lengths of stay. Even in severely shocked patients, acute internal fixation has been shown to be safe and may decrease 24-hour red cell transfusion requirements compared to staged approaches [8]. Beside the direct bleeding control of acute vascular bleeders, the most vital goal of early fixation is the reduction of the intrapelvic cavity volume. A 5-cm opening of the pelvic ring can increase its volume by 10% to 20%, whereas early approximation of the native anatomy facilitates tamponade of venous and bony bleeding and improves intrapelvic clot formation [5]. In addition, direct reconstruction of the pelvic ring offers further significant advantages in the treatment of polytrauma patients. Mechanical stabilization prevents undesirable movement at the fracture site, which significantly reduces the patients’ pain. Finally, achieving a stable pelvis early, particularly through minimally invasive “SI screws”, allows for easier in-bed rotation, patient positioning, and respiratory care in the ICU. It also simplifies necessary surgeries in other anatomical regions, such as the spine, which might otherwise be obstructed by external fixation frames [2].

It has been described that external fixation is generally considered insufficient for fixation of vertically unstable pelvic injuries [9]. Anteriorly placed external frames provide only marginal fixation for these posterior structures [9]. Clinical studies show that external fixators frequently fail to maintain reduction in type-C injuries, with one study reporting a failure rate in 38 out of 40 cases. During the period of fixation, vertical displacement of the posterior ring often increases significantly, for example, from an average of 8.9 mm to 15.2 mm [9, 10]. Moreover, external fixation carries a significant risk of pin-site infection, reported in approximately 24% of cases, which can complicate later definitive internal surgery [9, 10].

Emergency stabilization using a C-clamp is also not a safe alternative to external fixation. The high forces required to apply a C-clamp can lead to intra-pelvic penetration of the pins, over-reduction of the fracture, or over-compression of the pelvic ring. In cases of severe comminution or displaced fracture types, the device can paradoxically cause severe dislocations or sacroiliac displacement [3]. Clinical studies report that C-clamps can suffer from a loss of reduction and loosening in up to 13% of cases. Furthermore, pin malpositioning and device migration have been observed in approximately 5% of patients [3]. An initial application of a C-clamp significantly elevates the risk of infection for secondarily applied sacroiliac screws, with rates increasing from 3.2% to 20.8% leading to contraindications for surgical approaches and fixation techniques [3].

Recent literature demonstrates that early fixation of major fractures, such as unstable pelvic ring fractures, in polytrauma patients, generally defined as surgery performed within 24 h of admission, is a standard component of modern trauma management aimed at improving survival and functional outcomes [11]. Several publications have proven this approach to be safe and effective for severely injured patients [12, 13]. Minimally invasive fixation using SI Screws causes marginal soft tissue damage and low blood loss, thereby reducing the “surgical load” and helping avoid a physiological “second hit” in polytrauma patients. These screws can be initiated rapidly after admission—even in severely shocked patients—with studies showing they can be safely administered after arrival in the trauma bay [1, 3]. A significant benefit of SI screws is that they often serve as definitive fixation; while external fixators and C-clamps require a secondary “conversion” surgery in 100% of cases, properly placed rescue screws may eliminate the need for further posterior pelvic surgery [12].

### Limitations

This study has several limitations. The retrospective single-center design carries an inherent risk of selection and documentation bias. In addition, the small number of patients managed with external fixation limits the statistical power of all comparative analyses, and these results should therefore be interpreted with caution. Although the posterior pelvic ring was definitively stabilized during the index procedure, a subset of patients required staged anterior ring fixation as a separate secondary procedure, which contributes to the cumulative operative burden reflected in the total number of surgical procedures. Finally, radiographic outcome assessment was limited to the measurement of residual sacroiliac joint diastasis on postoperative imaging; postoperative loss of reduction and bony union were not systematically assessed and could not be reported. Future prospective studies should incorporate standardized radiographic endpoints.

In conclusion, our retrospective analysis of severely injured patients with unstable pelvic injury suggests that early definitive posterior fixation using percutaneous sacroiliac screws is a safe and feasible strategy for unstable pelvic ring injuries in Level-1 trauma settings, even for patients presenting in hemorrhagic shock. Further prospective studies are needed to investigate the exact effects of percutaneous techniques on outcomes in acute fixation in this patient group.

## Data Availability

No datasets were generated or analysed during the current study.

## Abbreviations

APC: Anterior–posterior compression
CM: Combined mechanism
ExFix: External fixator
InFix: Internal fixator
ISS: Injury severity score
LC: Lateral compression
LOS: Length of stay
SDS: Safe definitive surgery
SI: Sacroiliac
VS: Vertical shear
PI: Pelvic injury
